# Sero-prevalence of *Leishmania donovani* infection in labour migrants and entomological risk factors in extra-domestic habitats of Kafta-Humera lowlands - kala-azar endemic areas in the northwest Ethiopia

**DOI:** 10.1186/s12879-015-0830-2

**Published:** 2015-02-26

**Authors:** Wossenseged Lemma, Habte Tekie, Solomon Yared, Meshesha Balkew, Teshome Gebre-Michael, Alon Warburg, Asrat Hailu

**Affiliations:** Department of Medical Parasitology, School of Biomedical and Laboratory Sciences, College of Medicine and Health Sciences, University of Gondar, Gondar, Ethiopia; Department of Zoological Sciences, College of Natural Science, Addis Ababa University, Addis Ababa, Ethiopia; Department of Biology, College of Natural Science, Jigjiga University, Jigjiga, Ethiopia; Aklilu Lemma Institute of Pathobiology, Addis Ababa University, Addis Ababa, Ethiopia; Department of Microbiology and Molecular Genetics, The Institute for Medical Research Israel-Canada, The Kuvin Centre for the Study of Infectious & Tropical Diseases, The Hebrew University – Hadassah Medical School, The Hebrew University of Jerusalem, Jerusalem, 91120 Israel; Department of Microbiology, Immunology & Parasitology, Faculty of Medicine, Addis Ababa University, Addis Ababa, Ethiopia

## Abstract

**Background:**

Visceral leishmaniasis (VL) or kala-azar cases in seasonal labour migrants from highland areas are addressed to travel history to the Metema–Humera lowlands, northwestern Ethiopia. Factors that affect the incidence of VL in extra-domestic habitats were not evaluated. The aim of this study was to evaluate sero-prevalence of *Leishmania donovani* infection in randomly selected labour migrant workers and entomological risk factors which might affect the incidence of kala-azar.

**Methods:**

Sero-prevalence of *L. donovani* infection in labour migrants was obtained from Direct Agglutination Test (DAT) using blood samples. Logistic regression analysis was used to correlate the possible risk factors with *L. donovani* infection. The season for *L. donovani* infection or *Phlebotomus orientalis* bite was estimated from the study of population dynamic of *P. orientalis* in areas where the blood was sampled.

**Result:**

A total of 7, 443 *P. orientalis* (1,748 female and 5,695 male) were collected from agricultural fields and thickets of *Acacia seyal* using 461 CDC light traps. The highest mean number of *P. orientalis/*trap in the thickets of *A. seyal* and agricultural fields were 46.9 and 43.9 in March and April respectively. The mean *P. orientalis*/trap for November – May dry season in agricultural fields (11.39) and thickets of *A. seyal* (25.30) were higher compared to 0.66 in fields and 3.92 in thickets during June – August weeding season. Of the total 359 labour migrants screened using DAT, 45 (12.5%) were DAT-positive (≥1:800) for *L. donovani* infections. Very high titers (1:12800) were found in 3 (0.8%) individuals who had the risk of kala-azar development. Statistically significant p-values and odd ratio (OR) for staying in the areas both in the weeding and harvesting seasons (p = 0.035; OR = 2.83) and sleeping in the agricultural fields (p = 0.01; OR = 15.096) were positively correlated with *L. donovani* infection. Night harvest (p = 0.028; OR = 0.133) and knowledge about sign or symptoms (p = 0.042; OR = 0.383) were negatively associated with this infection.

**Conclusions:**

Sleeping in open agricultural fields was related with *L. donovani* infections in labour migrants during June-August weeding season.

## Background

Visceral leishmaniasis (VL) or kala-azar is a fatal systemic disease if left untreated [1,2]. There is high incidence of kala-azar in East Africa [3-5]; the second leading in annual incidence in the world, next to the Indian subcontinent [6]. Kala-azar distribution and incidence in East Africa are greatly influenced by environmental, behavioral and socio-economic factors in addition to the HIV co-infection and genetic susceptibility [7-12]. In East Africa and the Indian subcontinent, VL is caused by the *L. donovani* complex, unlike Europe, North Africa and Latin America where the agent is *L. infantum* [13,14]. Ethiopia has second largest number of annual VL cases (4000–7000) in Africa, next to Sudan [15]. In endemic areas of VL, *L. donovani* infection does not necessarily mean clinical illness (2). Due to the reasons not well understood, *L. donovani* infections remain asymptomatic in certain subjects and cause a lethal disease in others. The ratio of incident asymptomatic infections to incident clinical cases in Ethiopia is 5.6:1 (4) compared to the range from 1:2.6 to 11:1 in Sudan (3) and 4:1 in Kenya (5). Leishmanin skin test (LST) and direct agglutination test (DAT) are among the immunoassays widely used in kala-azar endemic areas to determine *L. donovani* infection rates (3–5). But, kala-azar patients will not show LST positive result until 3–6 months incubation phase becoming less useful as early diagnostic tool for infection detection during VL outbreaks [16]. Furthermore, asymptomatic subjects may have to be repeatedly exposed to the parasite before they undergo LST conversion [5]. Of the several serological tests, DAT appears to be a simple and economical test with high sensitivity and specificity [17]. However, it cannot differentiate among past kala-azar, subclinical infection, and active disease [18].

The largest kala-azar focus in Ethiopia is found in the Metema–Humera lowlands where Kafta-Humera is located [1]. The fertile black clay soil in the area is used for growing sesame, sorghum and cotton in a commercial scale. The agricultural activities (weeding and harvest) in Kafta-Humera lowlands attract around 200,000 seasonal labour migrants annually, mainly from the surrounding Amhara and Tigray highland areas [19]. In this region, kala-azar particularly affects migrant workers [1,19,20] in addition to residents involved in agricultural activities [20,21]. The population of Humera that were involved in agricultural activities were 45.6% positive for leishmanin skin test compared to 8.3% in non-farmers (urban and farm-owning population) with annual sero-conversion rate of 7% and less than 1%, respectively [20]. Kala-azar is a very important public health problem and causes high mortality and morbidity, especially among labour migrants. A total of 1, 258 VL cases were treated from 2009 – 2011 in Kasaye Abera Hospitals in Humera [19]. The aim of this study was to investigate the seroprevalence rate (DAT positivity and sero-reaction) of *L. donovani* infection in labour migrants and associated entomological risk factors in the extra-domestic habitats of the Metema–Humera lowlands.

## Methods

### Study area

Kafta Humera district is found in Western Tigray Region in northern Ethiopia. Humera town is the administrative center of the district. The town is situated near the borders of Sudan and Eritrea at 14°17′N latitude, 036°39′E longitude and 637 m elevation. The smaller towns near Humera include Rawyan (14°17′ 19″N, 036°37′ 18″E, 600 meter above sea level), May Kadra (14°08′ N, 036°34′ E, 612 m a.s.l) and Adebay (14°17′ 22 ″N, 036°38′E, 625 m a.s.l). Population dynamics of *P. orientalis* was conducted in extra–domestic areas (agricultural fields and associated thickets of *Acacia seyal*) around Adebay (site-1), Rawyan (site-2) and May Kadra (Gelanzeraf and Mysegen-Mehari areas) (site-3) from May, 2011 to June, 2012). The minimum distance of the sample sites from the adjacent town was not less than 10 km. In Kafta-Humera, November – May is a dry season while June-October is rainy season. The hottest month is May while August is the month with the highest rainfall. In the typical agricultural fields in Kafta-Humera lowlands, there are *Balanites aegiptiaca* trees at about 25 m intervals in any direction. The clear spaces between these trees are usually used for crowing sesame. After the land was ploughed and seeded in the mid and late June, labour migrants engaged in removing weeds from sesame seedlings, mostly after establishing themselves in the agricultural fields. Weeding of the sesame field is repeated during the flowering stage around August. Mostly, the same labour migrants perform the harvest and separation of the seed from the plant in September–October (harvest season) before their return to home in the highlands. But, some labour migrants return to their home at the end of August to take care of their own farm activities at home. Yet, there are some labour migrants who would come to the lowlands for September–October harvest. Only the seeds are removed from the agriculture fields. The agricultural leftovers are left on the field which serves as food for animals (cattle, sheep and goats) during dry season. Almost always, there are low laying areas (depressions), next to the agricultural fields, where water floods during rainy season. These depressions are the areas where thickets of *A. seyal* are found. Animals also graze inside these thickets. Labour migrants often go to these thickets for collecting fire woods. During the agricultural activities, all movements of the labour migrants are limited in the agricultural fields and thickets of *A. seyal*. The labour migrants who are stationed in the agricultural fields are mostly supplied with water and raw materials to make their own food. They cook and sleep under the shade of *B. aegiptiaca* trees*.* The big cracks in the agricultual fields and sparse thickets of *A. sayal* are the breeding habiatats of *P. orientalis* [22,23]. Rarely, labour migrants come from areas outside Amhara and Tigray region.

### Study design

Since all study subjects were not using bed nets, the effect of using bed net could not be evaluated. Only labour migrants with seasonal visit to the study area from June to November agricultural season were included in the study. Individuals with past kala-azar cases were excluded. During sampling, the research team patrolled through the different agricultural fields of the study sites for sampling of blood from volunteer migrant labour workers involved in harvest of sesame. Oral consent was obtained to obtain blood for testing by DAT, with a pre-planned sample size of 359. Sero-prevalence of *L. donovani* infection for labour migrants was obtained from Direct Agglutination Test (DAT) which would be correlated with the possible risk factors (Age intervals, address, number of visits, knowledge about the transmission of kala-azar, weeding and harvest stays, sleeping in the farm and night harvest) for *L. donovani* infection by calculating the odd ratio in logistic regression analysis. The season for *L. donovani* infection or *P. orientalis* bite was estimated from the study of population dynamic of *P. orientalis* in areas where the blood was sampled. Attempt to reach Mysegen-Mehari areas in October, 2012, where big farms were located, failed as the road for the car was out of use. As a result, blood sampling was conducted in all study areas including Mysegen-Mehari areas during the next season (October, 2013), after the reconstruction of the road.

### Popuation dynamics of *Phlebotomus orientalis* in agriculture fields and associated sparse thickets of *Acacia seyal*

CDC light traps were set at 6 p.m, hanged at about 0.5 meter above ground level, and left overnight till 6 a.m. Sand flies from traps were kept in 95% alcohol before transferring to physiological saline containing detergent for washing, sorting into genus *Sergentomyia* and *Phlebotomus* and counting before mounting in Hoyer’s medium for species identification [24-26]. The Mean Monthly Density (MMD) of P. orientalis was determined by total counts divided by the number of CDC light traps.

### Blood sampling

Blood samples were randomly obtained from labour migrants involved in harvest of sesame in the study area after oral consents were obtained. Blood sample were obtained by skilled laboratory technician from the forearm veins of the labour migrants using sterile needle and transferred to vacutainer tube which was allowed to clot at room temperature (25°C). Serum was separated by centrifugation (1200 cycle per minute) before it was stored at - 20°C. The cold serum box containing each serum sample was transported to the Department of Microbiology, Immunology and Microbiology in Medical faculty of the Addis Ababa University, where DAT was performed.

### Direct Agglutination test (DAT)

Serum samples were diluted in a dilution solution containing 0.9% NaCl solution, 0.1% (wt/vol) Fetal Calf Serum and 0.2 M 2-mercaptoethanol. A twofold dilution series of the sera was made, starting at a dilution of 1:100 until a maximum dilution of 1:12800. Prior to its use, aliquots of FD antigen (Royal Tropical institute *L. donovani* promastigote) (The Netherlands) were reconstituted in 5 ml of normal saline (0.9% [wt/vol] NaCl). Reconstituted antigen (50 ml) was added to each well of the microwell plate containing 50 ml of diluted serum. A 24-h incubation period at 18 to 20°C employed before the reading of the DAT. The DAT titers were grouped as negative (<1:100), reactive (1:100 to <1: 800), most probably infected (1:800–1:6400) and infected and at risk of kala-azar development (>6400 (12800)).

### Ethics

The study protocols were approved by the ethical review committee of the Department of Zoological sciences, Addis Ababa University and the Tigray Regional State Health Bureau. Each participant was involved after oral consent was given. The study subjects with DAT positive results were informed to go to the nearest kala-azar treatment center in Axum, Gondar or Humera for check up for any signs and symptoms of kala-azar during the follow up period (first 6 months) via their telephone numbers.

### Statistical analysis

Descriptive statistics was used to calculate the Mean and standard deviation of the socio- demographic characters and Mean Monthly Density (MMD) of *P. orientalis.* Logistic regression analysis (bivariate) was used to study the risk factors associated with kala-azar infections. For each of the study factors, risk was estimated by calculating the odds ratio (OR) as an approximation of the relative risk with 95% confidence intervals (CIs) using statistical package of social science (SPSS) version 20.

## Results

### Population dynamics of *Phlebotomus orientalis*

**Figure 1:**
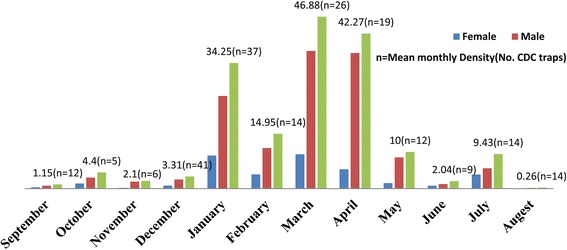
**Population dynamics of** ***P. orientalis*** **in tickets of** ***Acacia seyal*** **(n = 214) in Rawyian, Adebay, Baeker, Gelan Zeraf and Mysegen-Mehari during May, 2011 to June, 2012.**

A total of 7, 443 (1,748 females and 5, 695 males) *P. orientalis* was collected from agricultural fields (859 females; 2, 593 males; 3, 452 total) and thickets of *A. seyal* (889 females; 3, 102 males; 3, 991 total) in Adebay, Rawiyan, Gelanzeraf and Mysegen – Mehari using 461 CDC-light trap nights. In addition, *P. papatasi* (n=158), *P. duboscqi,* (n=42), *P. bergeroti,* (n=11), *P. rodhaini* (n=24) and *Sergentomyia spp.* (n=91, 292) were collected. The agricultural fields and thickets of *A. seyal* have similar sand fly fauna and both habitats are characterized by big cracks during November – May dry season. But, there were statistically significant differences (P = 0.000) among the mean densities of *P. orientalis* for the two habitats in different seasons and months. The number of *P. orientalis* is slightly higher in the thickets of *A. seyal* (Table 1)*.* Following the heaviest rain in August, the population of *P. orientalis* drops almost to zero. The number of *P. orientalis* remained low until January, the time for the beginning of high temperature and heavy cracking of the black soil (Figure 1). The mean density of *P. orientalis* during the November – May dry season was 11.39 **±** 22.98 in agricultural fields which was lower than 25.30 ± 40.06 in thickets of *A. seyal.* March is the month with the highest overall mean number of *P. orientalis* in thickets of *A. seyal* (46.88 ± 71.46). Significantly, high mean number of *P. orientalis* also collected from agricultural fields in April (43.89 ± 61.57). The lower mean number of *P. orientalis* in agricultural fields (0.03 ± 0.08) and tickets of *A. seyal* (1.97 ± 1.81) during June -August weeding season might have also been attributed to the strong wind, especially during May and June. Extremely low mean number of *P. orientalis* was obtained during September - October harvest season in agricultural fields (0.66 ± 0.65) and thickets of *A. seyal* (3.92 ± 7.71).

**Table 1:** **Total number of sand flies collected from Adebay, Rawiyal, Gelanzeraf and Mysegen – Mehari agricultural fields and thickets of** ***Acacia seyal*** **(May, 2011 to June, 2012)**

**Species**	**Agricultural fields**			**Thickets of** ***Acacia Seyal***		
	**No. CDC traps (n) = 247**			**No. CDC traps (n) = 214**		
	**Female**	**Male**	**Total**	**Female**	**Male**	**Total**
*Phlebotomus orientalis*	859	2593	3452	889	3102	3991
*P. papatasi*	34	67	101	18	39	57
*P. duboscqi*	2	27	29	0	13	13
*P. bergeroti*	0	6	6	0	5	5
*P. rodhaini*	17	3	20	3	1	4
*Sergentomyia* spp	25419	22631	48050	16337	26905	43242

### Sero-prevalence of *Leishmania donovani* infection in migrant workers

Overall 359 blood samples were obtained from labour migrants, of these 243 (67.7%) showed no reaction for direct agglutination test while the other 116 (32.3%) were seroreactives. In this study, titers from 1:200 to <1:800 were considered simply as reactive. Those with DAT results greater or equal to 1:800 titers 45 (12.5%) were considered as DAT positive study subjects (Table 2). The 42 Individuals (11.7%) with 1:800–1:6400 titers were most probably has *L. donovani* infections but did not have kala-azar. Only 3 serum samples (0.8%) had titers greater than 6400 (12800) depicting a high risk for development kala-azar.

**Table 2:** **Socio-demographic data and Knowledge, Attitude and Practice (KAP) of labour migrants related to kala-azar infection in the Kafta – Humera lowlands**

**Variables**	**Characterstics**	**Frequency**	**Percent**
1. Sample sites	Adebay	54	15
	Rawiyan	24	6.7
	Gelanzeraf	180	50.1
	Mysegen- mehari	101	28.1
	Total	359	100
2. Visit to Humera	First time	65	18.1
	Second time	79	22
	Third time	83	23.1
	More than three times	132	36.8
	Total	359	100
3. Knows at least a symptom of Kala-azar	no	185	51.5
	yes	174	48.5
	Total	359	100
4. Knows agent causing kala-azar	no	358	99.7
	yes	1	0.3
	Total	359	100
5. Knows at least a method to prevent kala-azar	no	290	80.8
	yes	69	19.2
	Total	359	100
6. Knows kala-azar is a vector-borne disease	no	345	96.1
	yes	14	3.9
	Total	359	100
7. Stayed both weeding and harvest season	no	101	28.1
	yes	258	71.9
	Total	359	100
8. Involved in night harvest activities	no	56	15.6
	yes	303	84.4
	Total	359	100
9. Sleep in the farm	no	68	18.9
	yes	291	81.1
	Total	359	100
10. DAT Positive (Direct agglutination test + ve)	no	314	87.5
	yes	45	12.5
	Total	359	100
11. Seropositive	no	243	67.7
	yes	116	32.3

### Risk factors

Only one woman was found during sampling of 359 migrant workers and all of them knew kala-azar as a disease. The age groups 15–24 and 25–34 accounted for 79.9 % of all the study subjects with range from 14–61 years. The study subjects came from Amhara region (75.2%), Tigray region (21.2%), Addis Ababa (0.8%) and Wolaita (2.9%) which are non-endemic to kala-azar. About 80% of the migrant workers visited the study area for the second time and above. Except one study subject, all participants did not know the agent causing kala-azar. Very few subjects (3.9%) knew kala-azar as a disease transmitted by insect (vectors). Comparatively, more participants (48.5%) mentioned at least a sign or symptom of kala-azar (Table 2). Until sesame was harvested, domestic animals were guarded in dense mixed forest far away from the farm. There were no animals in the farm including dogs during June - November. The strongest predictors for reporting DAT positivity and sero-reaction in labour migrants were sleeping in the farm under *B. aegiptiaca*, recording odds ratios of 15.096 and 6.63 respectively. This indicated that labour migrants who sleep in the farm were 15 times more likely to have DAT positive (*L. donovani* infection) or 6.63 more likely to have sero-reactive than those sleep in the camp, controlling all other factors in the model. Similarly, staying both in the harvest and weed season in Humera was 2.83 times more likely to have DAT positive (kala-azar infection) or 4.43 times sero-reactive than when labour migrants stayed in Humera only during the harvest time following heavy rain season in September – November (Table 3).

**Table 3:** **Results from logistic regression analysis to evaluate factors that affect the incidence of** ***Leishmania donovani*** **infection in the Kafta - Humera lowlands**

**Dependant variables**	**B**	**S.E.**	**Wald**	**df**	**Sig**	**Exp (B)**	**95.0% C.I. for EXP(B)**	
							**Lower**	**Upper**
A) DAT Positive								
- Stayed both weeding and harvest season	1.04	0.492	4.463	1	0.035	2.83	1.078	7.428
- Involved in night harvest activities	−2.014	0.918	4.816	1	0.028	0.133	0.022	0.806
- Sleep in the farm	2.714	1.048	6.71	1	0.01	15.096	1.936	117.716
- Age intervals	−0.263	0.226	1.358	1	0.244	0.769	0.494	1.197
- Address	0.92	0.42	4.785	1	0.029	2.508	1.1	5.717
- Number of visit to Humera	0.181	0.176	1.064	1	0.302	1.198	0.85	1.691
- Knows, at least, a sign or symptom	−0.961	0.473	4.13	1	0.042	0.383	0.151	0.966
- Knows agent causing VL	−18.573	40193	0	1	1	0	0	
- Knows, at least, a method to prevent VL	0.475	0.565	0.709	1	0.4	1.609	0.532	4.864
- Knows VL is a vector born disease	−0.468	1.146	0.167	1	0.683	0.626	0.066	5.916
- Constant	−4.664	1.264	13.627	1	0	0.009		
B) Sero-reaction								
- Stayed both weeding and harvest season	1.513	0.348	18.889	1	0.00	4.542	2.295	8.988
- Involved in night harvest activities	−1.778	0.767	5.375	1	0.02	0.169	0.038	0.76
- Sleep in the farm	1.892	0.779	5.901	1	0.015	6.635	1.441	30.541
- Age intervals of migrant workers	−0.304	0.158	3.679	1	0.055	0.738	0.541	1.007
- Address of the labour migrants	1.067	0.321	11.021	1	0.001	2.907	1.548	5.459
- Number of visit to Humera	0.248	0.131	3.588	1	0.058	1.281	0.991	1.655
- Knows, at least, a sign or symptom	−1.084	0.318	11.622	1	0.001	0.338	0.181	0.631
- Knows agent causing VL	24.004	40193	0	1	1	2.66	0	
- Knows, at least, a method to prevent VL	−0.248	0.418	0.352	1	0.553	0.78	0.344	1.77
- Knows VL is a vector born disease	−1.367	1.096	1.555	1	0.212	0.255	0.03	2.185
- Constant	−3.336	0.865	14.888	1	0	0.036		

The odds ratio of 0.133 for DAT positive and 0.169 for sero-reactivity for night harvest was less than 1, indicating involving in the night harvest was 0.133 times less likely to have DAT positive or 0.17 times less likely for sero-reactivity. Involving in night harvest during September – October might not be risky to have *L. donovani* infections as population is the lowest in these months.

## Discussion

Labour migrants have little knowledge about the agent, vector and ways to prevent kala-azar (P > 0.05). These results indicated the need for public health awareness. But, knowledge of signs and or symptom has showed negative correlation to DAT positivity or sero-reaction (p < 0.05) indicating a possible contribution of knowledge to personal protection. Where there is no utilization of bed nets, sleeping in the open farm or camp, labour migrants could be easy targets for *P. orientalis*. Sleeping in the farm was found 15 times more likely to have DAT positive or 6.63 more likely to have sero-reaction than those sleeping in the camp. The risk of kala-azar infection in labour migrants might have been exacerbated by poor dietary condition and lower educational attainment [19]. In general, the number of visits did not show association with DAT positive (p = 0.302) result or sero-reaction (p = 0.058). But, immunological naive labor migrants with first visit were expected to have a high risk of getting kala-azar.

Evidence has showed shift of *P. orientalis* from black cracking soil in agricultural fields and thickets of *A. seyal* to hollows in tree trunks in dense mixed forest near farms in July before the heaviest rain in August wiped out this vector [23]. This result could also suggest the shift of *P. orientalis* from their breeding habitats to possible shelters like grass huts of labour migrants in the camp which might have increased the chance of *P. orientalis* bites or *L. donovani* infection during the weeding season. Labour migrants and permanent residents who lived in tukuls with grass wall were found to be 4.5 times more likely to be exposed to kala-azar infection than those living in houses with mud-plastered walls [19]. Successful *P. orientalis* collections in May and June have been indicated [27] with the *P. orientalis* abundance increasing 20-fold from March-April to June in 1995/96 in Sudan. In our study, regression analysis has showed neither staying during September – October harvest nor night harvest were associated to *L. donovani* infection (Table 3). Thus, labour migrants could be exposed to *P. orientalis* bites in June - August weeding season before the heavy rain destroys *P. orientalis* in August. Peak monthly kala-azar cases in permanently settled farmers in Shiraro (northern Ethiopia) was reported in January (39/223 or 17.49%) during study period lasted from August, 2010 to July, 2011 (Hailu *et al*., unpub. data). Probably, July - August might be the season when these farmers, also, get the infection after possible 2–6 months incubation period suggested earlier [3]. The relative risk of *P. orientalis* bite in this habitat might have increased in July - August rainy season after the soil cracks were sealed off and *P. orientalis* shifted to the villages for protection. In eastern Sudan, transmission most likely occurs during the dry season (March to May) and illnesses often occur in October and November with incubation period from 2 to 6 months [3]. This might be true for pastoral communities who expose themselves to highest possible *P. orientalis* bites during their stay in *Acacia - Balanites* woodlands for guarding livestocks. Labour migrants in the Metema-Humera lowlands are not exposed to highest rates of P. orientalis bites expected in May as the majority normally arrive in the farms later in June or July.

## Conclusion

Labour migrants are most probably exposed to kala-azar infection during June - August weeding season. Staying during September – October harvest season or involving in night harvest might not associated with the risk of kala-azar infection due to the reduction of *P. orientalis* density.
